# Predictability and heritability of individual differences in fear learning

**DOI:** 10.1007/s10071-014-0752-1

**Published:** 2014-05-05

**Authors:** Jason Shumake, Sergio Furgeson-Moreira, Marie H. Monfils

**Affiliations:** Department of Psychology, The University of Texas at Austin, 108 E. Dean Keeton Stop A8000, Austin, TX 78712-1043 USA

**Keywords:** Individual differences, Artificial selection, Fear memory, Rat vocalization, Extinction learning

## Abstract

Our objective was to characterize individual differences in fear conditioning and extinction in an outbred rat strain, to test behavioral predictors of these individual differences, and to assess their heritability. We fear-conditioned 100 Long-Evans rats, attempted to extinguish fear the next day, and tested extinction recall on the third day. The distribution of freezing scores after fear conditioning was skewed, with most rats showing substantial freezing; after fear extinction, the distribution was bimodal with most rats showing minimal freezing, but a substantial portion showing maximal freezing. Longer rearing episodes measured prior to conditioning predicted less freezing at the beginning of extinction, but differences in extinction learning were not predicted by any baseline exploratory behaviors. We tested the heritability of extinction differences by breeding rats from the top and bottom 20 % of freezing scores during extinction recall. We then ran the offspring through the same conditioning/extinction procedure, with the addition of recording ultrasonic vocalizations throughout training and testing. Only a minority of rats emitted distress vocalizations during fear acquisition, but the incidence was less frequent in the offspring of good extinguishers than in poor extinguishers or randomly bred controls. The occurrence of distress vocalizations during acquisition predicted higher levels of freezing during fear recall regardless of breeding line, but the relationship between vocalization and freezing was no longer evident following extinction training, at which point freezing levels were influenced only by breeding and not by vocalization. The heritability (*h* ^2^) of extinction recall was estimated at 0.36, consistent with human estimates.

**Electronic supplementary material:**

The online version of this article (doi:10.1007/s10071-014-0752-1) contains supplementary material, which is available to authorized users.

## Introduction

Fear conditioning is a form of classical conditioning in which an animal acquires an association between an aversive unconditioned stimulus (US) and a previously neutral conditioned stimulus (CS) through CS–US pairings. In laboratory studies of fear conditioning with rodent models, the aversive US is often a brief electric current delivered to the feet through a metal grid floor, and the CS is usually a tone or a light. Conditioned fear is then measured by the ability of the CS to evoke a conditioned emotional response (CER) such as freezing. Fear *extinction* is learning to inhibit the expression of a CER through repeated presentations of the CS by itself, without the US.

Typically, these and other Pavlovian processes are assumed to be universal and invariant—common to all organisms and operating according to fixed laws. Any variability in behavior not due to treatment effects is usually regarded as noise, and any subject that, for example, fails to condition or fails to extinguish becomes an outlier to be ignored. In the best case, this traditional “mean behavior” approach may indeed reveal typical cognitive processes that function more or less the same across most individuals. In the worst case, it may lead to an ecological fallacy: if different animals recruit fundamentally different cognitive processes in response to identical conditioning procedures, the mean behavior of these animals may be an artificial composite that fails to represent the real functioning of any individual animal. Historically, the latter possibility has been widely ignored despite strong evidence that individual animals often respond very differently to the same conditioning procedures, which has been known since the beginning of classical conditioning research.

In fact, Pavlov (1927) outlined extensive evidence that “different types of nervous systems” manifest as different temperaments with differential conditionability. For example, he identified an “excitable type” of dog that readily acquires excitatory CRs, but only acquires inhibitory CRs with great difficulty, “as if the animals’ nervous system opposes a barrier to their establishment” (Pavlov 1928, p. 107). However, the emerging field of behaviorism apparently ignored Pavlov’s admonishment that “the type of nervous system of the individual animal must never be ignored” (Pavlov 1928, p. 110), because there was a dearth of empirical investigation in this area for several decades.

Only in the past decade has the study of individual differences in animal behavior emerged as a key target of research (Réale et al. 2010; Wolf and Weissing 2012). However, despite this and a rich history of human research linking personality and conditionability dating back to the 1950s (Eysenck 1955), the burgeoning movement of animal personalities has made little headway into animal research on fear conditioning. To date, only one study has detailed the distribution of individual differences in acquisition and extinction learning in a large sample of rats (Bush et al. 2007). This study used Sprague–Dawley albino rats, and our first objective was to perform a similar analysis using a large sample of Long-Evans hooded rats.

A common question regarding individual differences is the extent to which they reflect inherited traits. Artificial selection, or selective breeding, is a well-established method for demonstrating the heritability of learning ability (Wahlsten 1972). For example, avoidance learning, which combines classical fear conditioning and operant conditioning, showed a large response to selection in the very first generation of what would later become the Roman High and Low Avoidance lines (Bignami 1965), with an estimated heritability of 56 % (Wahlsten 1972). Interest in the behavioral genetics of Pavlovian fear conditioning is, however, a surprisingly recent development. Radcliffe et al. (2000) were the first to demonstrate that the acquisition of conditioned freezing in mice responded to selective breeding with an estimated heritability of 37 %.

The heritability of the *acquisition* of conditioned freezing has now been replicated by applying selective breeding to mice (Ponder et al. 2007, 2008; Choi et al. 2012) and rats (De Castro Gomes and Landeira-Fernandez 2008). There have also been several reports of strain differences in fear extinction (Hefner et al. 2008; Chang and Maren 2010) and differences in fear extinction that emerge after artificial selection for some other trait (Sartory and Eysenck 1976; Shumake et al. 2005; Ponder et al. 2007; Muigg et al. 2008). However, to our knowledge, no one has attempted to directly select for freezing differences following fear extinction after matching animals for post-acquisition levels of freezing. Such differences are important to understand because individual differences in fear extinction appear far more pronounced than individual differences in fear acquisition (Burgos-Robles et al. 2007, 2009; Peters et al. 2010) and may be more relevant to modeling susceptibility and resilience to anxiety disorders, especially PTSD (Holmes and Singewald 2013). This is because, while differential acquisition of fear memory may play an important role in the development of these disorders, fear acquisition itself is not pathological; rather, the source of dysfunction is the stubborn persistence and intrusion of fear memories. Thus, persistent fear after extinction may be the conditioning endophenotype that is most relevant to these disorders.

Here, we report the results of a selective breeding experiment for both increased and decreased freezing 24 h after fear extinction in Long-Evans rats. We chose Long-Evans hooded rats because they are a widely available outbred strain commonly used in behavioral research, and we crossed rats from different commercial suppliers to maximize the genetic diversity of our founding population. Compared with albino rats, Long-Evans rats appear more anxious in the elevated plus maze (Adamec 1997) and show greater spontaneous recovery after fear extinction (Chang and Maren 2010), suggesting that it may be easier to select for resistance to fear extinction within the Long-Evans strain. While we were also interested in selecting for rapid fear extinction, our priority was to find rats that would fail to extinguish. We chose not to cross with an albino strain because previous work has shown that the allele that confers albinism is itself associated with reduced fear conditionability (Ponder et al. 2008), and selecting for such a mechanism of fear resilience would not have translational relevance for non-albino populations. In addition, we looked for predictors of fear conditioning and extinction differences in terms of exploratory activity and ultrasonic vocalizations (USVs), and we report a heritability estimate for fear extinction based on the first generation of an ongoing artificial selection experiment.

## Methods

### Overview

50 male and 50 female Long-Evans hooded rats were purchased from Charles River Laboratories and Harlan Laboratories, respectively and used as the founding population (Generation 0). They arrived in our animal facility at 6 weeks of age and were same-sex housed in pairs. Following 1 week of acclimation, rats were phenotyped for exploratory activity, fear conditionability, and fear extinction ability, as described in the Phenotyping section below. As described in the Selection Criteria section below, three breeding lines were generated by crossing males and females from the two different suppliers. Selective breeding was applied to two of the lines, and an unselected control line was also created from the same Charles River/Harlan cross. The offspring (Generation 1) underwent the same fear conditioning/extinction paradigm, with the addition of sound recordings to quantify ultrasonic vocalizations (USVs), as described in the Phenotyping section below. All procedures followed US National Institutes of Health guidelines and were approved by the University of Texas Institutional Animal Care and Use Committee.

### Phenotyping

Each generation was phenotyped for fear extinction. In addition, Generation 0 males were phenotyped for exploratory activity, and Generation 1 males and females were phenotyped for USVs.

#### Fear conditioning and extinction

Rats underwent fear conditioning and extinction at approximately 60 days of age in a Coulbourn operant box controlled by Graphic State software. On Day 1, following 10 min of habituation to the conditioning chamber, rats received 3 conditioning trials of a tone (5 kHz for 20 s) co-terminating with a foot shock (0.7 mA for 0.5 s) separated by a variable inter-trial interval (ITI) of 1–3 min. On Day 2, rats received 18 extinction trials of tone-alone presentations of the same duration and ITI as experienced in acquisition. On Day 3, rats received 3 memory-recall trials of tone-alone presentations, again with the same time parameters. Rats were videoed by overhead cameras, and the videos were later scored by two independent raters who timed seconds of freezing behavior. The raters were blind to the genetic lineage of the subjects. The intra-class correlation coefficient (ICC) for agreement between the two raters across all observations was 0.94 ± 0.01 (95 % confidence limits).

#### Exploratory behavior

Rat exploratory behavior was tested prior to fear conditioning and extinction in a Med Associates activity chamber (43.2 × 43.2 × 30.5 cm) consisting of clear plastic sides and a white Plexiglas floor. This apparatus detects horizontal and vertical motion via two parallel grids of intersecting infrared beams (one at floor level and one at a height that the rat can only reach by jumping or rearing up on its hind legs). A linked computer records the rat’s X,Y,Z coordinates every 50 ms. Rats were tested in two different setups: (1) an open-field setup, consisting of direct placement in the bare apparatus as described above and (2) a light–dark setup, in which a dark Plexiglas box with a rat-sized doorway covers exactly half of the chamber. Rats were tested for 10 min each day for 2 days for each setup (for a total of 40 min over 4 days), and the order of the setups was counterbalanced. Days 1 and 2 of the open field were conducted identically: the rat was placed in a corner of the chamber and allowed to move freely. Days 1 and 2 of the light–dark test were conducted differently: on Day 1, the rat was restricted to exploring the dark box; on Day 2, the rat was placed in the dark box, but allowed to move freely between dark and light compartments. Luminance (outside of the dark box) was provided by overhead incandescent lights adjusted to provide 100 lux at the level of the chamber.

#### Vocalizations

Vocalizations were recorded as WAV files using Avisoft-RECORDER (Version 4.2, Avisoft Bioacoustics, Berlin) with a sampling rate of 250,000 Hz, 16 bit format. Custom-written Python programs (available at https://github.com/EliMor/LabScripts) applied a fast Fourier transform (FFT) length of 4,096 samples to locate and extract the 20 s segments of the files corresponding to the frequency of the tone CS with an additional 20 s before each tone onset and after each tone offset. These 1-min clips were then imported to Avisoft SASLab Pro (Version 5.2, Avisoft Bioacoustics, Berlin), which was used to create spectrograms and detect calls with an automatic threshold-based algorithm. Call detection by the computer was verified by a trained observer and, if necessary, manually adjusted to eliminate false positives. The duration of each call was automatically measured along with peak amplitudes and frequencies, and these data were labeled as occurring before, during, or after the CS. Calls were categorized according to frequency range as indicating either negative (18–32 kHz) or positive (32–96 kHz) affect. In addition, reaction to the shock itself was measured as the energy of the large-amplitude component of a pain-induced vocalization, characterized by the simultaneous production of a wide range of frequencies, both audible and ultrasonic, without sharp fundamental frequencies (Jourdan et al. 1995)—in informal terms, a “shriek” or “scream”. As this occurs simultaneously with any other noise the rat makes in response to the shock (e.g., jumping up and down on the grid floor), we are technically measuring the energy of *all* sounds produced in response to the shock, but the pain-induced vocalization is the predominant one. This sound energy, measured in decibels (dB), increases linearly with shock intensity (Levine et al. 1984), which we confirmed with our own apparatus and methods (Online Resource 1).

### Selection criteria

Founding breeders for the two selected lines were chosen by a two-step procedure. First, selection for both lines was restricted to subjects showing robust freezing 24 h after acquisition. Second, subjects from this subsample were ranked according to their freezing scores 24 h after extinction training, and the top and bottom 10 males and females (each comprising 20 % of the screened sample) were chosen as the founders of the low extinguisher (LE) and high extinguisher (HE) lines. The optimal cutoff criterion for “robust freezing” was set at 75 %, the value which achieved minimal differences between groups post-conditioning relative to maximal differences between groups post-extinction. (See “Determination of cutoff criterion” under “Data analysis” section below.)

The rationale for this two-part criterion is that differences in freezing behavior after extinction are a function of both differences in extinction learning and differences “carried over” from acquisition learning, and we wanted as much as possible to select for differences in extinction in the absence of differences in acquisition. Heritable differences in fear acquisition have already been established with selective breeding experiments (Radcliffe et al. 2000; Ponder et al. 2007, 2008; De Castro Gomes and Landeira-Fernandez 2008), and we wished to control for this factor in assessing whether differences in extinction learning have a heritable component. Moreover, differences in initial conditioned freezing can be generated by the incidental selection of arbitrary factors unrelated to emotional learning, such as general activity level or sensitivity to the tone or shock. By matching both lines in terms of fear conditionability, we circumvented these potential confounds without the need for an extensive battery of sensory and motor tests to rule them out. In other words, if the lines are equivalent in their initial freezing response to a fear-conditioned tone, they must have similar abilities to hear the tone, feel the shock, and inhibit motor activity.

A randomly bred (RB) control line was started from a random selection of 10 males and 10 females from the remaining 60 % of the initially screened sample that were not chosen for the selected lines. Since it excluded population extremes, the selection of Generation 0 of the RB line was only partially random, but the selection of subsequent generations was entirely random.

### Husbandry

Breeding pairs were housed together (1 male to 1 female) for 10 days (i.e., the average length of 2 estrous cycles). Females were then rehoused with their former female cage mate for the next 10 days, and then single housed until giving birth. On the day after their birth, newborns were briefly separated from their mothers for sexing and culling. The number of males and females were counted, and litters were reduced in size to 12 pups (ideally to 6 males and 6 females or to the most equal sex ratio possible). The litters were then left undisturbed except for weekly cage changes until weaning at 21 days. From weaning until 41 days, rats were group housed with all of their same-sex siblings, usually in groups of 6. Thereafter, rats were housed with same-sex siblings in groups of 2–3. Rooms were maintained at steady temperature (21 ± 1 °C) and a 12–12 light–dark cycle (lights on at 7:00 and off at 19:00). Food and water were provided ad libitum.

### Data analysis

Data were evaluated with appropriate tests of statistical significance as described in the Results. Statistics were computed in R version 3.0.1 and SPSS version 21. In this section, we describe in greater detail the machine learning approach we developed for selecting optimal breeders and the metrics from quantitative genetics we used for estimating heritability.

#### Determination of cutoff criterion for post-acquisition freezing

Normally, artificial selection studies use a single metric from a single time point as a criterion for breeding, or a simple aggregate measure (sum or average) from multiple time points. We, however, were faced with the unique challenge of selecting for two behaviors from two time points that are inherently at odds with one another, that is, we wanted our groups to show the same behavior at Time 1, but very different behavior at Time 2. Because behavior at Time 1 is correlated with behavior at Time 2, this results in a loss–gain tradeoff: the more similar the animals are at Time 1 (gain), the less divergent they are at Time 2 (loss); the more divergent they are at Time 2 (gain), the less similar they are at Time 1 (loss). Thus, we were faced with the problem of finding the optimal matching criterion to minimize loss and maximize gain in this scenario. To obtain this value, we mathematically formalized the loss as follows.

A cost function was written in MATLAB that, given a cutoff value of *θ* (% freezing after acquisition), (1) filters out cases less than *θ*, (2) selects the top and bottom 10 subjects based on freezing scores after extinction, and (3) computes the cost of this classification, *J*(*θ*), as follows

$$J\left( \theta \right) = \frac{{1 + ({\text{LE}}_{\text{acq}} - {\text{HE}}_{\text{acq}} )^{2} }}{{({\text{LE}}_{\text{ext}} - {\text{HE}}_{\text{ext}} )^{2} }}$$

where LE and HE are the mean freezing scores of the low and high extinguishers, respectively, at 24 h after acquisition (subscript acq) or extinction (subscript ext). Thus, the cost is minimized by decreasing the group mean difference post-acquisition and increasing the group mean difference post-extinction. The addition of the constant 1 to the numerator insures that post-extinction group differences continue to be maximized in the event that pre-extinction group differences are minimized to zero. The cutoff value of *θ* was then determined by iteratively re-computing the cost of *θ* on the interval of 1–85 %, which was the 60th percentile of the distribution or the maximum number of subjects that could be excluded and still yield 10 breeding pairs for each selected line. For females, optimal classification was achieved at *θ* = 76–77 %. For males, optimal classification was achieved over *θ* = 67–85 %. Since the optimal cutoff interval for the males encompassed that of the females, the cutoff value for the female sample was applied to both sexes. Thus, subjects chosen as founders for the LE and HE lines were required to have a post-acquisition (pre-extinction) freezing score greater than 75 %.

#### Estimation of heritability

Heritability (*h* ^2^) was estimated as the slope of a weighted linear regression of offspring mean on parent mean across all lines. Regression weights were determined by the following formula (Falconer and Mackay 1996):

$$W_{i} = \frac{{n_{i} + n_{i} T}}{{1 + n_{i} T}}$$

where the weight (*W*) for the *i*th family is proportional to the number of offspring tested (*n*) and *T*, defined as follows:

$$T = \frac{{t - 0.5b^{2} }}{1 - t}$$

where *b* is the slope of the unweighted regression and *t* is the intra-class correlation, defined as follows:

$$t = \frac{{{\text{MSA}} - {\text{MSW}}}}{{{\text{MSA}} + \left( {n_{\text{o}} - 1} \right){\text{MSW }}}}$$

where MSA is the mean square among litters, MSW is the mean square within litters, and

$$n_{\text{o}} = \bar{n} - \mathop \sum \nolimits \frac{{(n_{i} - \bar{n})^{2} }}{{\left( {k - 1} \right)N}}$$

where *n*_*i*_ is the number of individuals in the *i*th litter, *k* is the total number of families, *N* is the total number of offspring, and $\bar{n}$ is the average family size.

#### Response to selection

We established a criterion metric for categorizing rats as belonging to extreme phenotypes (see first section of “Results” below). Response to selection was then evaluated by testing whether breeding resulted in a significant change in the binomial proportion of observed phenotypes as evaluated with exact binomial tests. For the HE line, we evaluated the directional hypothesis that the proportion of HE phenotypes would increase from the expected incidence in the general population. For the LE line, we tested the opposite.

## Results

### Generation 0

#### Distributions of individual differences in fear acquisition and extinction

**Fig. 1:**
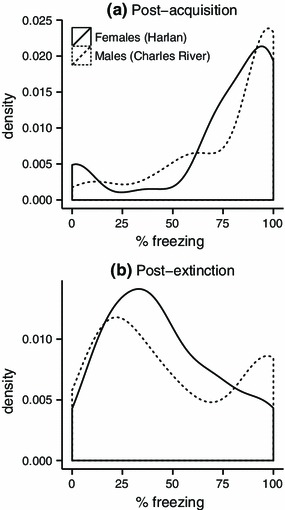
Probability density plots of the distributions of median freezing scores from the first 3 trials of the extinction session (**a** “Post-acquisition freezing”) and from the 3 probe trials conducted 24 h later (**b** “Post-extinction freezing”). Separate distributions are shown for 50 males obtained from Charles River and 50 females obtained from Harlan

Figure 1 shows the sample distributions for freezing after fear acquisition and fear extinction for males and females from different animal suppliers. The distribution for tone-conditioned freezing after fear acquisition showed highly negative skew, with the mass of the distribution concentrated toward maximum freezing for both males and females. The distribution for fear extinction for males was bimodal with a primary peak just below 25 % freezing and a secondary peak at maximum freezing, whereas the distribution for females was closer to normal with a central tendency around 35 % freezing, but with an extended right tail.

Based on these distributions, we also characterized the animals as discrete “types”. A robust acquisition/robust extinction type was defined as freezing 75 % or more during at least 2 of the first 3 trials of extinction training (24-h fear recall) and 25 % or less during at least 2 of the 3 recall trials at 24 h post-extinction. A robust acquisition/negligible extinction type was defined as freezing 75 % or more for at least 2 of the 3 trials at both time points. Based on these criteria, 20 % of rats (11 males + 9 females) were robust learners of both acquisition and extinction (HE phenotype), and 20 % of rats (14 males + 6 females) showed robust fear acquisition with negligible fear extinction (LE phenotype). (The remainder includes 30 % of rats that showed robust fear conditioning with intermediate levels of extinction, plus 29 % of rats that did not show robust fear conditioning.)

#### Extinction curves for HE versus LE phenotypes

**Fig. 2:**
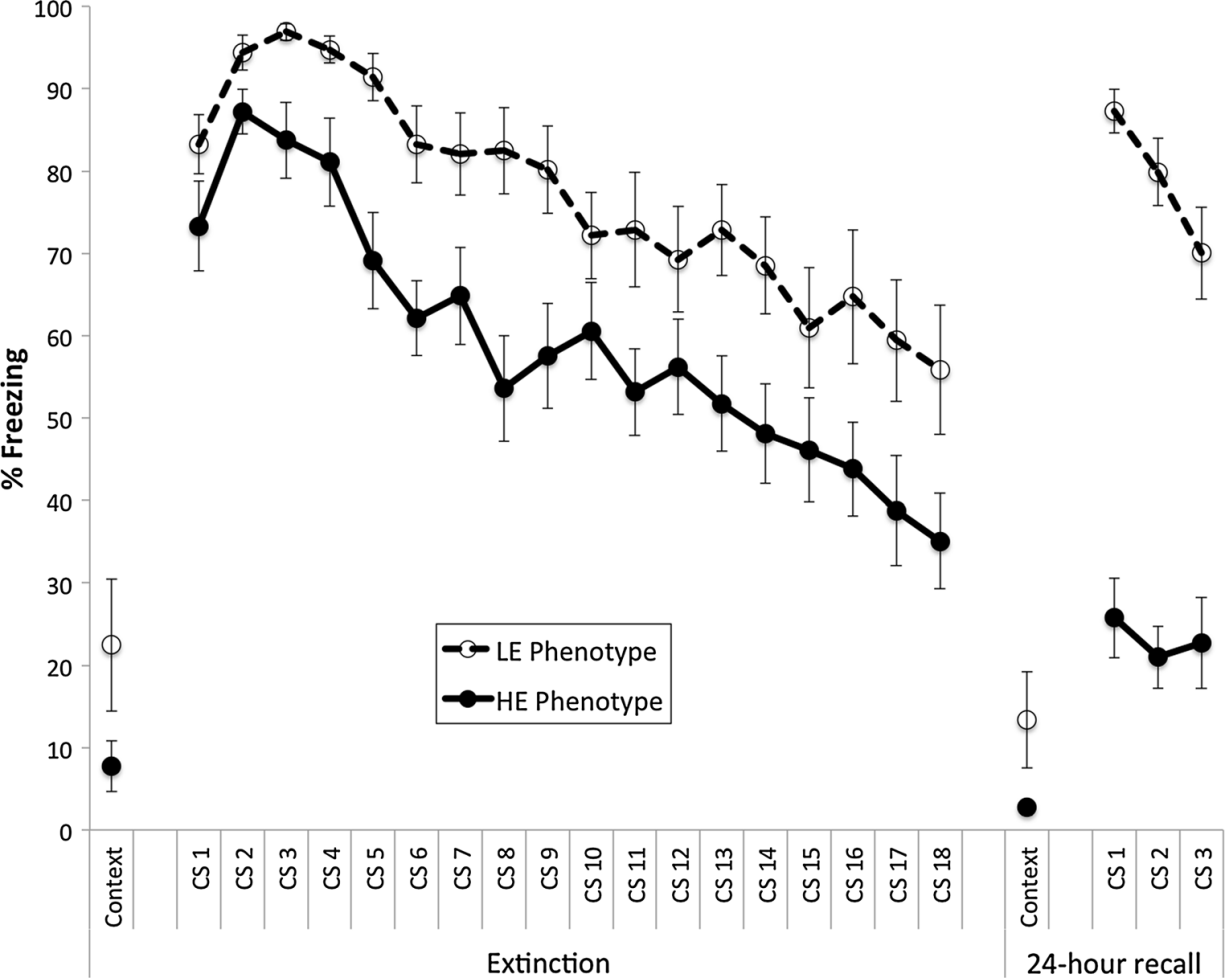
Extinction curves for rats classified as high (HE Phenotype) versus low (LE Phenotype) extinguishers based on differences at 24-h recall. Freezing to context was not significantly different between the two phenotypes, but there was a significant difference in freezing throughout extinction (repeated measures main effect, *P* < .001)

For rats classified as HE or LE (see above), conditioned freezing was scored for all 18 trials of the extinction session to assess whether the classification based on 24-h post-extinction reflected a failure to extinguish in the first place versus a failure to recall extinction learning. In addition, we scored conditioned freezing to context prior to the delivery of the first CS. As shown in Fig. 2, subjects classified as LE tended to show somewhat higher freezing throughout the extinction session, including freezing to context though the latter was not significantly different, *t*(38) = 1.71 and 1.79, *P* = .12 and .15 (*P* values calculated using bootstrap method because of unequal variances and the presence of floor effects). However, analyzing the 18 extinction trials as repeated measures, the overall difference in freezing was statistically significant, *F*(1,38) = 17.9, *P* < .001, but the *rate* of extinction learning, reflected by the slopes of the extinction curves (the phenotype × trials interaction), was not significantly different between phenotypes, *F*(17,646) = 0.627, *P* = .87.

Thus, despite our best effort to match individual HE and LE subjects in terms of acquisition levels of freezing, when averaged together, there are small but significant group differences in freezing that are maintained over the course of extinction. However, these differences pale in comparison with those observed at 24-h recall. An analysis of covariance (ANCOVA) showed that freezing during the last three trials of extinction does not predict freezing at 24-h recall, *F*(1,37) = 0.96, *P* = .34, and the effect of phenotype on 24-h recall remains highly significant even after adjusting for freezing differences during extinction, *F*(1,37) = 7.81, *P* = .008. In summary, rats divided into HE and LE phenotypes based on a post-extinction recall test also showed similar, but much smaller, group mean differences during the extinction session itself.

#### Relationship to exploratory activity

Prior to fear conditioning and extinction, male subjects were tested for 4 days in exploratory chambers that automatically measured horizontal (ambulatory) and vertical (rearing) activity. Rats were tested under either an open-field condition or a light–dark condition, which were counterbalanced in terms of sequence. This allowed us to measure activity in response to a relatively threatening environment (the open field) versus a relatively safe environment (the dark box). Supporting this threat versus safety distinction, fecal boli counts were more than twice as high in the open-field condition (*M* = 4.7) as in the light–dark condition (*M* = 1.8). In addition, each test condition was repeated to allow us to measure activity in response to novelty (Day 1) versus familiarity (Day 2). In the case of the light–dark condition, the rat was restricted to the dark box on Day 1 to obtain a direct contrast measure between a novel open field and a novel covered field; on Day 2, the rat was permitted to exit the dark box if it chose, so this day provided the traditional metrics (latency to exit dark box and time spent in light box) of the light–dark test (Crawley and Goodwin 1980) in addition to the total activity metrics, which consisted of absolute horizontal and vertical activity (ambulatory distance and rearing counts, respectively) and time-normalized versions of the same metrics (velocity and rearing duration, respectively). Velocity reflects the vigor of movement (distance in cm covered per second of time), and rearing duration reflects the mean length of a single rear (seconds spent rearing per rearing count).

As the first step in an exploratory analysis to assess predictive relationships between the activity variables and the variables measured during and after fear conditioning and extinction, a factor analysis with principal components extraction and varimax rotation was used to reduce the activity data from 18 measurements to 6 dimensions (eigenvalues > 1) explaining 76 % of the total variance. The correlations of each factor with the original measurements are shown in Table 1, with the original variables clustered according to their primary factor loadings. Factor 1 primarily reflects activity level in the open field, with positive loadings of both ambulatory distance and rearing frequency measures from both novel and familiar sessions of the open field. Ambulatory velocity from the light–dark test also had a moderate negative loading on this factor. Factor 2 primarily reflects the willingness to explore the light compartment in the light–dark emergence task, as indicated by increased time spent in the compartment and a reduced latency to enter it. Greater ambulation and rearing counts in the light–dark test also contribute to this factor. Factor 3 reflects stable differences in the duration of individual rearing episodes as observed across all testing conditions. Factor 4 reflects measurements from the dark condition (in which the rat was confined to the dark box) and is comprised of higher activity levels (ambulation and rearing) coupled with slower velocity of movement. Factors 5 and 6 were limited to movement velocity in the novel and familiar open fields, respectively, indicating these measurements were independent of each other as well as all of the other activity metrics. In general, the factor analysis suggested three conclusions: (1) absolute rearing and ambulatory counts tend to co-vary within testing condition but are independent across testing conditions; (2) individual differences in rearing duration reflect a trait that persists across testing conditions; and (3) velocity is dependent upon environmental novelty and largely independent of all other metrics.

**Table 1:** Correlations of individual activity measures with their principal components

Activity measure	Principal component					
	1	2	3	4	5	6
Ambulatory distance, novel open field	.86					
Ambulatory distance, familiar open field	.80					
Rearing frequency, familiar open field	.77					
Rearing frequency, novel open field	.76					
Velocity, light/dark	−.54					
Voluntary time spent in light, light/dark		.91				
Ambulatory distance, light/dark		.85				
Latency to exit dark, light/dark		−.85				
Rearing frequency, light/dark		.76				
Rearing duration, novel open field			.87			
Rearing duration, familiar open field			.81			
Rearing duration, dark			.78			
Rearing duration, light/dark			.56			
Velocity, dark				−.84		
Ambulatory distance, dark				.67		
Rearing frequency, dark				.52		
Velocity, novel open field					.92	
Velocity, familiar open field						.93

Correlations with absolute value <.50 are omitted from table

Next, bivariate correlations were computed between factor scores corresponding to these 6 dimensions and freezing scores at 24-h fear recall and 24-h extinction recall (Table 2). For fear recall, correlations were based on the entire sample; for extinction recall, analysis was restricted to subjects showing robust (>75 %) initial fear conditioning. Only one significant relationship was found: longer rearing episodes predicted less freezing following fear acquisition, but not following extinction. A possible trivial explanation for this relationship is that rats predisposed to long rearing episodes were more likely to be rearing at the time they were shocked, which could have reduced the salience of the US. To assess this possibility, we examined videos from the acquisition sessions and made note if a rat was rearing during delivery of the US. This was only true for two rats and happened on only one of the three acquisition trials for each rat. Dropping these subjects from the analysis did not change the significance of the result.

**Table 2:** Correlations (*r*) between dimensions of exploratory behavior and conditioned freezing

	Post-acquisition freezing	Post-extinction freezing
1. Open-field activity	.26	−.06
2. Light-zone activity	.09	−.27
3. Rearing duration	−.56*	−.10
4. Dark-zone activity	.20	−.02
5. Velocity in novel open field	.18	−.04
6. Velocity in familiar open field	.01	−.07

* Correlation is significant at the 0.05 level after Bonferroni correction

In addition, we assessed those subjects classified as HE or LE for group mean differences on these behaviors. HE and LE rats were equivalent on every activity measure in every test condition (data shown in Online Resource 2), with a trend for longer exit latencies and less light-zone exploration for LE rats in the light–dark test, in line with the weak (nonsignificant) negative correlation observed between light-zone exploration and post-extinction freezing. However, neither difference was significant, *P* = .38 and .13 (exact two-tailed significance of the Mann–Whitney test statistic) for exit latency and light-zone exploration, respectively. Thus, rats classified as HE or LE appeared similar in terms of their baseline exploratory activity.

### Generation 1

A total of 314 offspring were born (LE = 102, HE = 109, RB = 103), and a total of 236 were tested (LE = 99, HE = 97, RB = 40). The difference in *N* from birth to testing reflects the culling of litters larger than 12 in the case of LE and HE rats. To save on time and housing costs, testing of RB subjects was limited to the 20 males and 20 females randomly selected to perpetuate the line. Four subjects (1 RB female, 1 HE female, 1 LE male, and 1 HE male) were excluded from data analysis because of equipment failure during training. In addition, one subject (1 HE male) was excluded from USV analysis because of a corrupted audio file.

#### Heritability and response to selection

**Fig. 3:**
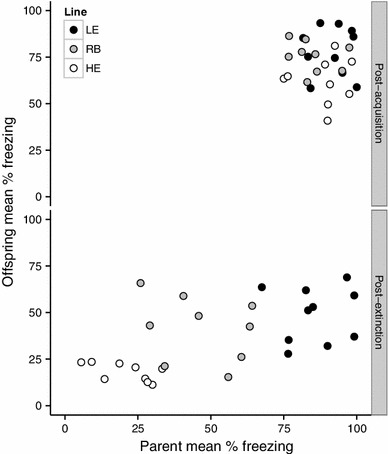
Scatter plots of offspring conditioned freezing as a function of their parents’ conditioned freezing 24 h post-acquisition and 24 h post-extinction. Each point represents a single family with the parent mean plotted on the X axis and the offspring mean plotted on the Y axis

**Fig. 4:**
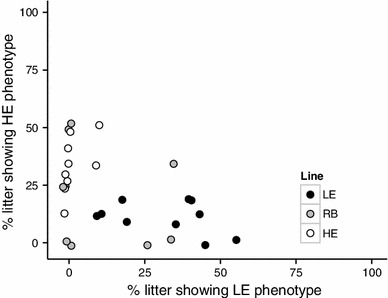
Scatter plot showing the composition of each litter in terms of the percentage of offspring showing an LE phenotype (>75 % freezing before extinction and >75 % freezing after extinction) versus the percentage showing an HE phenotype (>75 % freezing before extinction and <25 % freezing after extinction). Note that the axes do not represent freezing scores. Rather, they reflect the percentage of offspring in each litter that actually met the specified criteria of being LE or HE, regardless of whether they were born to an LE or HE parent. Each *point* represents a litter. The *shading* indicates whether the parents were LE, HE, or RB (see legend). The *X coordinate* indicates what percentage of the litter actually displayed an LE phenotype, and the *Y coordinate* indicates what percentage of the litter actually displayed an HE phenotype. This *graph* illustrates the variability in the response to selection: in any given litter, some rats show the phenotype of their parents, some show the opposite phenotype, and the remainder are somewhere in between

Individual differences in freezing behavior post-extinction were significantly heritable (Fig. 3), with an estimated *h* ^2^ of 0.36 ± 0.10 (*P* = .001). The offspring means for tone-conditioned freezing after fear acquisition were 77 % (SD = 26 %) for the LE line, 74 % (SD = 22 %) for the RB line, and 65 % (SD = 27 %) for the HE line. The means after fear extinction were 49 % (SD = 30 %) for the LE line, 41 % (SD = 25 %) for the RB line, and 18 % for the HE line. These group means suggest that heritability in the first generation was driven by a response to selection in the HE line rather than in the LE line. However, when the data are evaluated in terms of percentages of each litter belonging to extreme types (Fig. 4), offspring from LE parents had a 31 ± 6 % probability of meeting LE phenotype criteria (>75 % freezing both before and after extinction) and only an 11 ± 4 % probability of meeting HE phenotype criteria (>75 % freezing before extinction and <25 % freezing after). Exact binomial tests indicate that this represents a significant increase (*P* = .006) in the proportion of LE phenotypes from the expected 20 % in the founding population, and a significant decrease (*P* = .01) in the proportion of HE phenotypes. Offspring of HE parents had a 34 ± 4 % probability of meeting HE phenotype criteria and only a 2 ± 6 % probability of meeting LE phenotype criteria. This also represents both a significant increase in the incidence of the HE phenotype (*P* < .001) and a significant decrease in the incidence of the LE phenotype (*P* < .001). The RB offspring had a 20 ± 6 % probability of meeting HE criteria (exactly the same proportion as the founding population) and a 13 ± 9 % probability of meeting LE criteria. Given the smaller sample size and the absence of a directional hypothesis of phenotypic change for the RB line, the 13 % proportion of LE phenotypes is within the expected sampling variance of a true proportion of 20 % (*P* = .32). In summary, the incidence of desired phenotypes was increased in both of the selected lines, and there was no evidence of spontaneous drift in the RB line.

#### Vocalizations

**Fig. 5:**
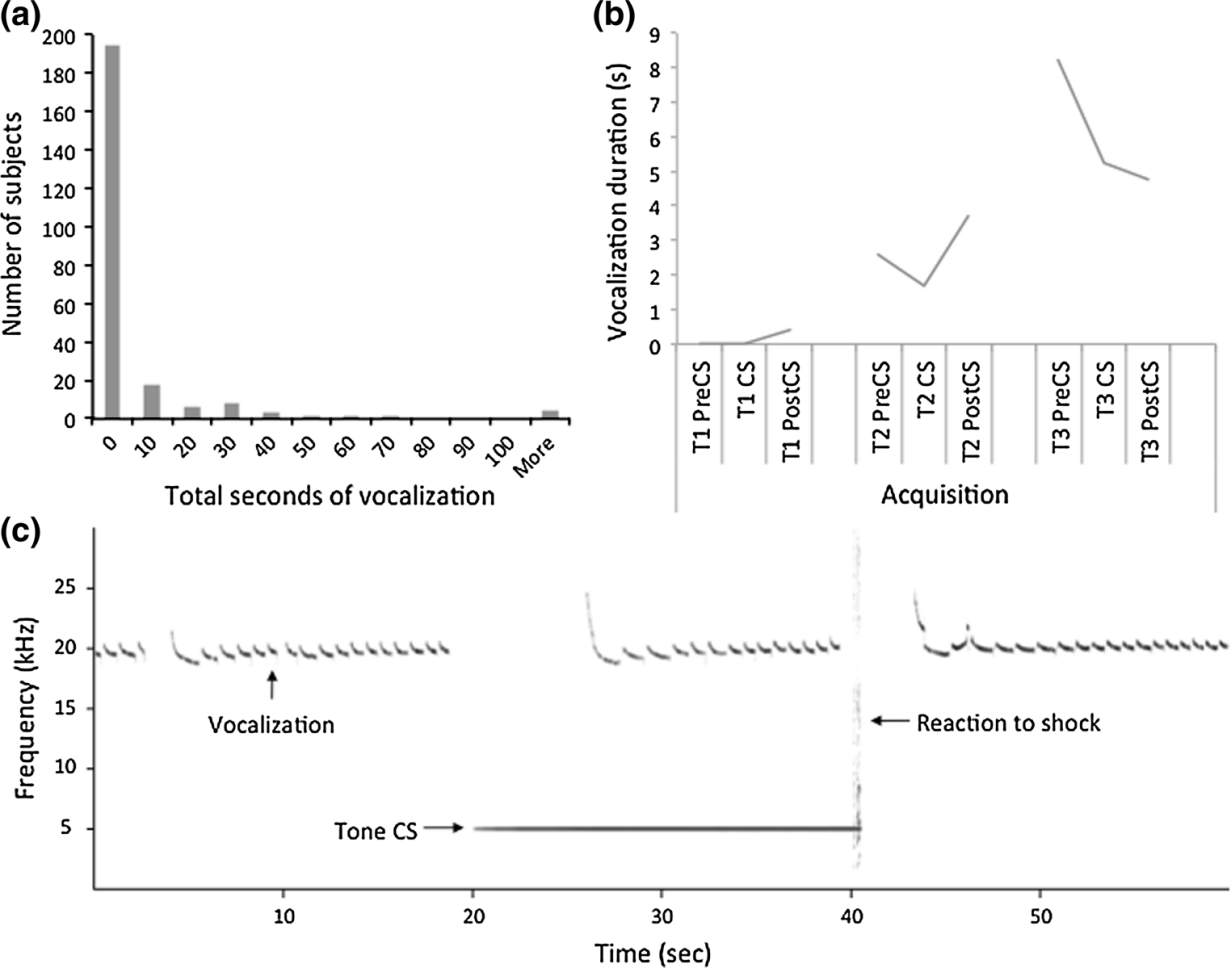
**a** Histogram showing frequency of 18–32 kHz “negative affect” vocalizations. **b** Mean 18–32 kHz vocalizations of the vocalizer sample (excluding non-vocalizers) as a function of training. Each trial (*T1*, *T2*, *T3*) is subdivided into three 20-s intervals before, during, and after the tone CS (Pre-CS, CS, and Post-CS). **c** Spectrogram from a single vocalizing subject for a single acquisition trial showing vocalizations at approximately 20 kHz, the 5 kHz tone CS, and the animal’s reaction to the shock US, which appears as a *vertical line* of broad-spectrum energy at the end of the tone

USVs were classified according to two categories and analyzed separately: lower frequency vocalizations of long duration (18–32 kHz, 300–4,000 ms), indicative of negative affect, and higher frequency vocalizations of short duration (32–96 kHz, 30–50 ms), indicative of positive affect. USVs were sampled in 1-min intervals centered at each tone CS presentation, and further categorized as pre-CS (occurring during the 20 s preceding a tone CS), CS (occurring during a 20 s tone CS), or post-CS (occurring during the 20 s following a tone CS). An example spectrogram is shown in Fig. 5c.

##### 18–32 kHz “negative affect” vocalizations

Given the highly skewed distribution of vocalizations concentrated at 0 (Fig. 5a), rats were first classified as vocalizers or non-vocalizers based on whether they vocalized for at least 300 ms (the minimum duration of one vocalization for this frequency band as recognized in the literature) at any time point during the acquisition or extinctions sessions. The incidence of vocalization differed significantly between the selected lines, with fewer HE rats classified as vocalizers than LE or RB rats, χ^2^ (2, *N* = 231) = 9.216, *P* = .01. There was also a significant sex difference in the incidence of vocalization, with fewer females vocalizing than males, χ^2^ (1, *N* = 231) = 7.112, *P* = .01. For females, there was no significant difference between selected lines, χ^2^ (2, *N* = 231) = 1.444, *P* = .56, but there was a significant difference between selected lines for males, χ^2^ (2, *N* = 231) = 7.187, *P* = .03. Table 3 shows the incidence of 18–32 kHz vocalizers within breeding line and sex.

**Table 3:** Incidence of vocalizations as a function of frequency (kHz), sex, and lineage

	Males	Females
*18*–*32* *kHz*		
Low extinguishers	23 % (*N* = 11/48)	8 % (*N* = 4/50)
Randomly bred	30 % (*N* = 6/20)	11 % (*N* = 2/19)
High extinguishers	5 % (*N* = 2/39)	4 % (*N* = 2/55)
*32*–*96* *kHz*		
Low extinguishers	23 % (*N* = 11/48)	32 % (*N* = 16/50)
Randomly bred	50 % (*N* = 10/20)	42 % (*N* = 8/19)
High extinguishers	38 % (*N* = 11/39)	22 % (*N* = 12/55)

**Fig. 6:**
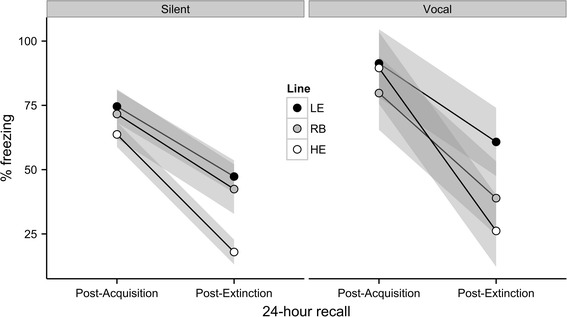
Freezing as a function of heredity, vocalization, and training. Breeding line is indicated by *shading* (see legend), and vocalization group (silent vs. vocal in emitting ~22 kHz sounds during acquisition) is indicated by *separate panels*. Means are plotted as *lines* with 95 % confidence bands. Vocalizers showed significantly more freezing after acquisition, but not after extinction. Breeding lines were significantly different after extinction, but not after acquisition, *p* < .05

Among those animals that did vocalize during the acquisition session, the vocalizations were induced by fear conditioning. As shown in Fig. 5b, USVs did not occur prior to delivery of the first shock and increased with each acquisition trial. The occurrence of vocalizations during acquisition predicted higher levels of freezing during fear recall 24 h later regardless of breeding line (Fig. 6). There was a significant main effect of vocalization, *F*(1,225) = 6.94, *P* = .009, with no significant main effect of breeding, *F*(2,225) = 1.02, *P* = .36, or interaction between vocalization and breeding, *F*(2,225) = 0.68, *P* = .51. However, this relationship between vocalization and freezing was no longer evident following extinction training. Freezing levels at this time point were influenced only by breeding, *F*(2,225) = 11.95, *p* < .001, and not by vocalization *F*(1,225) = 0.99, *P* = .32, or an interaction between vocalization and breeding, *F*(2,225) = 1.98, *P* = .14. In summary, the development of 18–32 kHz USVs during acquisition predicted greater levels of conditioned freezing before, but not after, extinction training.

##### 32–96 kHz “positive affect” vocalizations

Rats were first classified as vocalizers or non-vocalizers based on whether they vocalized for at least 30 ms (the minimum duration of one vocalization for this frequency band as recognized in the literature) at any time point during the acquisition or extinction sessions. The incidence of vocalization differed significantly between the selected lines, with more RB rats classified as vocalizers than LE or HE rats, χ^2^ (2, *N* = 231) = 6.532, *P* = .04. There was no significant sex difference in the incidence of vocalization, χ^2^ (1, *N* = 231) = 0.021, *P* = .88. The line difference was only significant when subjects were pooled across sex, and not significant within sex (*P* = .22 for females and .08 for males). Table 3 shows the incidence of 32–96 kHz vocalizers within breeding line and sex. Most of these vocalizations tended to occur toward the end of the extinction and extinction recall sessions (data not shown).

#### US reactivity

The delivery of the US was accompanied by broad-spectrum sound in the spectrogram (Fig. 5c), corresponding mainly to a pain-induced vocalization in response to foot shock. Over the range of 0–1 mA, the energy of this shock-evoked sound (measured in dB) increases as a highly linear function of shock amperage (data shown in Online Resource 1). Accordingly, we used this as a measure of the animal’s aversive reaction to the US, which was calculated as the change in dB from the second before versus the second after the onset of foot shock. Animals showed a wide range of sound energy increases in response to the shock, ranging from a minimum increase of 1 dB to a maximum increase of 48 dB above background, with a median increase of 10 dB. The metric was not normally distributed, showing positive skew and high kurtosis. According to Spearman’s rho, this metric was weakly related to the level of freezing both after fear acquisition, *r*_*s*_ (*N* = 231) = .23, *P* < .001, and after fear extinction *r*_*s*_ (*N* = 231) = .18, *P* = .005, with greater reactivity to shock predicting greater levels of freezing. The median sound increase for the selected lines was 11 dB for the LE line (range 1–39 dB), 10 dB for the RB line (range 1–48 dB), and 9 dB for the HE line (range 1–29 dB). Analyzing all three groups with a Kruskal–Wallis test, there was no significant difference among groups, *χ* ^*2*^ (2, *N* = 231) = 4.01, *P* = .13.

## Discussion

### Large individual differences in extinction recall

Prior to this study, there has been only one published study characterizing the distribution of individual differences in fear extinction learning in rats. Bush et al. (2007) combined data from vehicle control rats (Sprague–Dawley albinos) from several experiments to generate distributions of freezing scores before and after extinction. In contrast to the normal distribution of post-acquisition freezing scores reported by Bush et al. (2007), we found a negatively skewed distribution with a much higher mean level of freezing. A similarly skewed distribution of conditioned freezing scores was also reported for mice (Wehner et al. 1997) and is consistent with the finding that conditioned fear shows heterosis (Connor and Winston 1972), meaning increased phenotypic expression after the out-crossing of inbred lines. Both skewed distributions and heterosis suggest dominant gene action and are taken as evidence that a trait has tended to increase natural fitness in the population (Connor and Winston 1972). This makes sense because, if an aversive US meets a certain threshold of intensity, developing fear and avoidance would seem to provide an unequivocal survival advantage.

In contrast, we showed wide-ranging variance in freezing scores following extinction, with males showing a bimodal distribution. Bimodal distributions in extinction recall have also been reported in several studies from the Quirk laboratory using Sprague–Dawley rats (Burgos-Robles et al. 2007, 2009; Peters et al. 2010) as well as in a study using mice (Herry and Mons 2004). The exception to findings of bimodality is the study by Bush et al. (2007), who found an approximately normal distribution of freezing scores following extinction, albeit with a mean and standard deviation virtually the same as that found in this study.

The wide range of freezing scores following extinction suggests the existence of two competing phenotypes in the gene pool—one which favors original fear learning versus one which favors more recent learning when determining the response to a stimulus that has conflicting associations with both danger and safety. This also makes sense in that it is easy to imagine scenarios in nature in which either strategy would prove alternately adaptive or maladaptive, such that neither would emerge as a clear-cut winner over the other. This is consistent with models showing that the evolution of animal personalities or behavioral syndromes is favored by “conditions of intermediate ecological favorability” (Luttbeg and Sih 2010).

For example, if an animal survives an encounter with a predator, it will develop a conditioned fear response to any novel stimuli that occurred in temporal proximity to the predator. Subsequently, if the animal encounters one of these stimuli in the absence of a predator, it could mean one of two things. Either the initial pairing of the stimulus with the predator was coincidental, and the stimulus actually has no predictive value regarding the predator; or the stimulus does have predictive value, but its relationship to the predator is imperfect: sometimes it signals the predator, and sometimes it does not. The more innocuous exposures the animal has to the stimulus, the more likely the first possibility becomes, but it is never certain. Thus, the animal that resumes foraging in the presence of the stimulus risks being killed by the predator. On the other hand, the animal that continues to hide in response to innocuous stimuli risks missing out on safe feeding and mating opportunities. In the case of such an intermediate risk of predation, the strategy that confers the greatest chance of survival depends on the availability of survival resources, e.g., food and mates (Luttbeg and Sih 2010). If resources are scarce, the bold phenotype is favored; if resources are abundant, the cautious phenotype is favored. However, if resource availability is also intermediate, both phenotypes will persist, which is expected to increase the fitness of the population as a whole. This is because a population that harbors both types “averages out” its chances of survival, buffering itself against environmental fluctuations in predation pressure and habitat resources (Wolf and Weissing 2012).

In conclusion, more large-sample individual differences studies are needed using different strains and species to assess whether the skewed, low-variance distribution for conditioned fear and the bimodal, high-variance distribution for extinguished fear are replicable consequences of an evolutionary process converging on a single best strategy in one case and a behavioral-type strategy on the other.

### Selection of behavioral extremes in extinction recall

While our groups were selected to minimize differences at the beginning of extinction and maximize differences during 24-h extinction recall, we did not select for differences in extinction learning itself. Thus, the selected differences in long-term extinction memory could have emerged during the extinction session. However, the groups showed approximately parallel extinction curves. Although the low extinguisher group showed somewhat higher levels of freezing at the end of extinction training, this difference did not statistically account for the extreme divergence in freezing scores at extinction recall, on which the selection was based.

Our curves bear a striking resemblance to those reported by Burgos-Robles et al. (2007) after they also divided subjects based on low and high fear during extinction recall. Clearly, the differences observed at extinction recall are not simply a continuation of the previous day’s learning curves. Rather, the selected phenotype is characterized primarily by a rapid spontaneous recovery of fear, either from reduced consolidation of the extinction memory or a failure to retrieve it. This would be consistent with the findings of Norrholm et al. (2011), who hypothesized that the extinction deficits observed in PTSD patients are due to two independent mechanisms: exaggerated fear at the onset of extinction and insufficient fear inhibition to fully extinguish fear responses.

### Longer rearing episodes predict less conditioned freezing

Correlational analysis generally revealed no relationship between pre-conditioning measures of exploratory activity in the open field and light–dark tests. In particular, general activity level (locomotion + rearing) did not correlate with freezing either before or after extinction. This is an important negative finding in that individual differences in freezing levels cannot be explained by a propensity to be more or less physically active outside of the home cage. Interestingly, however, we did observe a significant inverse relationship between conditioned freezing and the average duration of a rearing episode in the open field and light–dark tests, but only after initial conditioning and not after extinction.

Rearing counts or frequencies are commonly reported measurements from the open field, and there is an old literature linking increased rearing frequency with superior avoidance acquisition and emotional learning in general (Lát 1965, 1967), presumably because the frequency of what Lát called the “standing-up reaction” reflects higher levels of “nonspecific excitability.” Sartory and Eysenck (1976) showed that the difference in avoidance learning between high and low-rearing rats stems largely from a differential reaction to shock: rats with a low incidence of rearing tended to cling to the grid during administration of shocks while rats with a high incidence of rearing tended to minimize contact with the grid, resulting in more shock exposure for low-rearing rats. However, this is unlikely to be a factor in simple fear conditioning because the shock duration is too brief for a clinging versus jumping reaction to influence exposure. Moreover, it was not rearing frequency that predicted conditioned freezing in our rats, but rather rearing duration: the average length of a single rearing episode.

Rearing duration has rarely been assessed in the literature. However, a few rat studies have linked shorter rearing episodes to hyperactivity brought about by genetic selection (Aspide et al. 1998), maternal separation (Colorado et al. 2006), or high doses of cocaine (Verheij and Cools 2011). However, in these studies hyperactive rats also showed increased rearing counts, such that decreased rearing duration was collinear with increased rearing frequency. In the PCA of the activity data reported here, rearing frequency loaded together with ambulatory counts and was specific to the apparatus the animal was being tested in (open field, dark box, or the light–dark choice condition), whereas rearing duration emerged as a distinct component that remained stable across all testing conditions. Rearing duration thus appears to be reflecting something other than hyper- versus hypo-activity. We can only conclude from the present data that the typical duration of a rat’s rearing episodes reflects a stable trait such that rats predisposed to longer rearing episodes are less likely to display passive defensive behavior in the form of freezing in response to a CS. This would be consistent with observations that rearing can be readily conditioned as an escape-from-fear response that leads to better long-term reductions in freezing (Cain and LeDoux 2007). Whether or not rats with long rearing episodes are less conditionable on other fear metrics, such as conditioned suppression, and the mechanisms underlying this association are questions meriting further inquiry.

### The ability to extinguish fear shows moderate heritability

While other studies have demonstrated the heritability of conditioned contextual freezing through selective breeding experiments with mice (Ponder et al. 2007, 2008) and rats (de Castro Gomes and Landeira-Fernandez 2008), to our knowledge we are the first to demonstrate heritability of extinction learning through selective breeding. To our knowledge, only one twin study (Hettema et al. 2003) has attempted to derive a heritability estimate for fear conditioning and extinction in humans. The best fitting model from this study arrived at the same estimate of heritability for extinction of a conditioned exciter that we did: 36 %. The fact that we arrived at exactly the same number is likely coincidental, but it is encouraging that two different methods of inheritance estimation using data from two different species would converge on a similar value.

It is important to note that, unlike heritability estimates derived from twin studies, estimates derived from parent–offspring regression do not control for the influence of shared early environments. Thus, our heritability estimate could reflect genetic inheritance, epigenetic inheritance, early shared environment, or some combination thereof. For rats in particular, maternal care is a well-established vector of non-genetic inheritance that influences many metrics of stress reactivity (Meaney 2001). In other words, perhaps HE rats were licked more by their mothers than LE rats, and this, rather than genetic inheritance, accounts for their superior extinction ability. To definitively rule out this possibility we would need to conduct a cross-fostering experiment (have LE rats raised by HE mothers and vice versa). However, although maternal care influences contextual fear conditioning, it does not affect tone fear conditioning, either in terms of acquisition or extinction (Bagot et al. 2009). Since we selected breeders based on freezing to tone, not context, this finding suggests that HE and LE mothers should have been equivalent in terms of maternal care.

Finally, could the fear conditioning procedure itself have induced epigenetic modifications that were passed on to the offspring? Although it is possible for the effects of stress exposures to be passed on to subsequent generations, this phenomenon has only been demonstrated for severe or chronic stressors, such as maternal deprivation and malnutrition (Gapp et al. 2014). While fear conditioning has been shown to result in epigenetic modifications, these appear to play a dynamic role in memory formation and are localized to specific neural circuits (Miller and Sweatt 2007). It is not evident that the transient stress associated with fear conditioning (in this case, exposure to 3 brief foot shocks and 21 subsequent presentations of stimuli that once predicted foot shock) is capable of modifying the DNA of germ cells, which would be required for direct epigenetic inheritance. Even if this were the case, all of the parents received exactly the same amount of stress, so this factor alone cannot explain the mean difference between the offspring of HE and LE parents.

### Vocalizations reflect breeding and predict conditionability

Among male Wistar rats, there was a high incidence of negative affect USVs during acquisition (5/7 rats vocalizing at 0.5 mA and 7/7 rats vocalizing at 0.8 mA) (Wöhr et al. 2005). By comparison, our Long-Evans rats were much less likely to vocalize in general. For example, at an intensity of 0.7 mA, only 30 % of male RB rats vocalized during acquisition. This may suggest that Long-Evans rats as a strain are less likely to show ~22 kHz vocalizations during fear conditioning than albino rats. Supporting this conclusion, one study has shown that Long-Evans rats do indeed vocalize significantly less than Sprague–Dawley rats during tone-shock conditioning (Graham et al. 2009). However, this study also found that Long-Evans females vocalized more than Long-Evans males, whereas our present study found that males vocalized more than females.

Despite the relatively low incidence in vocalizations in our subjects overall, the incidence among HE rats was especially low (4–5 %) and significantly lower than LE or RB rats. Because these differences in vocalizations occurred during the acquisition session, they suggest that genetic factors governing acquisition may play a role in whether an animal is ultimately able to extinguish a CS–US association. One should note that this effect on acquisition occurred despite our efforts to match the initial founders of the LE and HE lines in terms of post-acquisition levels of freezing. On the one hand, this suggests that the genetic influences on acquisition and extinction processes are overlapping. On the other hand, our results also show that the effect of selective breeding on USVs during acquisition is independent of its effect on extinction of conditioned freezing. That is, among vocalizing subjects, there is still a significant difference in extinction between the HE and LE lines. Indeed, this difference becomes even more apparent when differences in vocalization are controlled for. Regardless of breeding line, the volume of sound emitted as an acute reaction to shock and the duration of ultrasonic distress vocalizations emitted throughout the acquisition session predicted higher levels of subsequent conditioned freezing. This suggests that individual differences in the appraisal of the US are a significant factor underlying conditionability.

## Conclusion

In conclusion, we have found evidence that certain factors related to exploratory style (rearing duration) and emotional expressivity (vocalizations in response to stress) may influence an animal’s ability to acquire fear associations and/or modify the way in which this fear is expressed (e.g., freezing vs. active coping). In addition, to borrow Pavlov’s terminology, there appear to be two “types of nervous systems” when it comes to consolidating and/or retrieving fear extinction: one that favors the original fear learning and one that favors subsequent safety learning. Moreover, these phenotypes are moderately heritable, as demonstrated by a response to selection after only one generation of breeding. This selection response appears to have tapped into factors governing the initial impact of fear conditioning as well as separate factors specific to the process of extinction. In this context, it is interesting to note that the inheritance data from the twin study on fear conditioning and extinction (Hettema et al. 2003) was best explained by two latent genetic factors: one associated more with acquisition of conditioned exciters, and the other associated more with extinction and the acquisition of conditioned inhibitors. However, the influences of these factors showed considerable overlap as well. Henderson (1968) also found that inheritance of acquisition versus extinction of a CER in mice was largely independent in terms of genetic correlation. Further generations of selective breeding will determine whether the abilities to acquire and extinguish fear are ultimately dissociable in terms of genetic inheritance.

## Electronic supplementary material

Below is the link to the electronic supplementary material.

Supplementary material 1 (PDF 84 kb)

Supplementary material 2 (PDF 111 kb)
