# Supplementary material for: Predictability and heritability of individual differences in fear learning
Source: Anim Cogn. 2014 May 5;17(5):1207–21. doi: 10.1007/s10071-014-0752-1 (PMC4138434; doi:10.1007/s10071-014-0752-1)

Title: Predictability and Heritability of Individual Differences in Fear Learning
Journal: *Animal Cognition*
Authors: Jason Shumake, Sergio Furgeson-Moreira, and Marie H. Monfils
Affiliation: Department of Psychology, The University of Texas at Austin
E-mail: shumake@utexas.edu

Online Resource 1

In our study we measured the sound energy emitted by rats in response to electric shock as an index of shock reactivity, under the assumption that the loudness of this reaction increases as a function of perceived shock intensity. To validate this assumption, we administered 4 shocks of varying intensity (0.25, 0.5, 0.75, or 1.0 mA) to 8 adult Long-Evans rats (4 males + 4 females), each of equal duration (0.5 seconds) separated by a fixed 1-minute interval. We then took the dB reading associated with each shock and performed linear regressions for each subject as a function of shock intensity. The plots of each regression and the associated r^2 are given in this figure. The 0 mA point on the x-axis corresponds to the baseline dB reading in the absence of shock and reflects the level of background noise. Note that, while there are individual differences in the sound energies for a given shock intensity, each subject shows a highly linear relationship between sound energy and shock intensity. This supports the use of sound energy as a metric for aversive reactions.

Title: Predictability and Heritability of Individual Differences in Fear Learning
Journal: *Animal Cognition*
Authors: Jason Shumake, Sergio Furgeson-Moreira, and Marie H. Monfils
Affiliation: Department of Psychology, The University of Texas at Austin
E-mail: shumake@utexas.edu

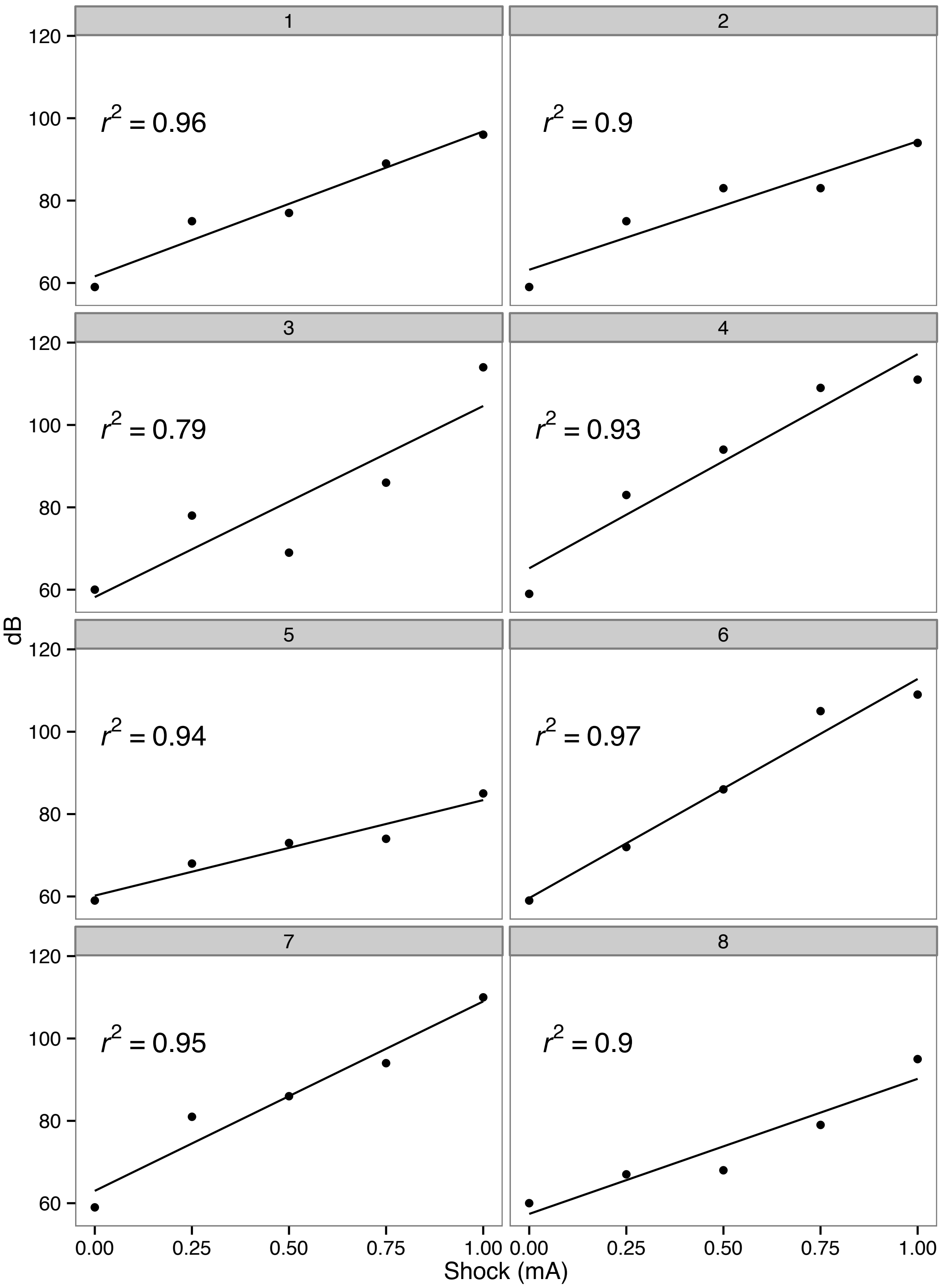

Supplement: Supplementary file 1 — Supplementary material 1 (PDF 84 kb) [file 10071_2014_752_MOESM1_ESM.pdf]
