# Supplementary material for: Predictability and heritability of individual differences in fear learning
Source: Anim Cogn. 2014 May 5;17(5):1207–21. doi: 10.1007/s10071-014-0752-1 (PMC4138434; doi:10.1007/s10071-014-0752-1)

Title: Predictability and Heritability of Individual Differences in Fear Learning
Journal: *Animal Cognition*
Authors: Jason Shumake, Sergio Furgeson-Moreira, and Marie H. Monfils
Affiliation: Department of Psychology, The University of Texas at Austin
E-mail: shumake@utexas.edu

Online Resource 2

Boxplots of activity and anxiety metrics of rats from the founding population classified as high extinguishers (HE) and low extinguishers (LE) across varying testing conditions: (a) ambulatory distance, (b) ambulatory velocity, (c) rearing frequency, (d) rearing duration, e) latency to exit the dark box, with failure to exit assigned a maximum score of 600 s, and f) the time spent exploring the light box. In the “Dark” condition, rats were restricted to a dark box. In the “Light/Dark” condition, rats were placed in the same dark box and given free access to an adjoining open, illuminated compartment. In the “Open Field” conditions, the dark box was removed and rats were placed in an open, illuminated compartment. This test was performed twice: “Novel” and “Familiar” conditions. No significant differences were found.

Title: Predictability and Heritability of Individual Differences in Fear Learning
Journal: *Animal Cognition*
Authors: Jason Shumake, Sergio Furgeson-Moreira, and Marie H. Monfils
Affiliation: Department of Psychology, The University of Texas at Austin
E-mail: shumake@utexas.edu

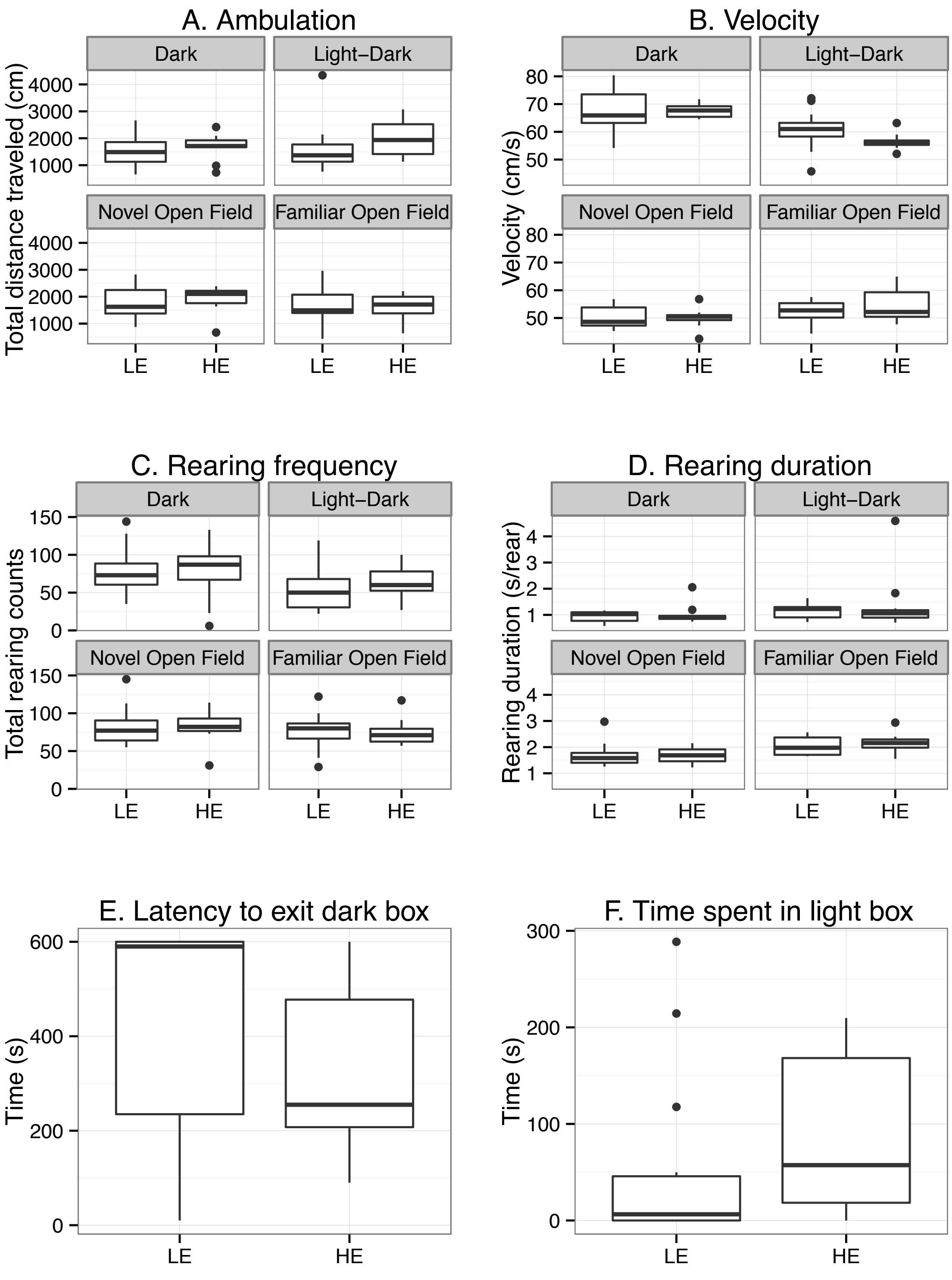

Supplement: Supplementary file 2 — Supplementary material 2 (PDF 111 kb) [file 10071_2014_752_MOESM2_ESM.pdf]
